# Graded epistemic justification

**DOI:** 10.1007/s11098-020-01512-0

**Published:** 2020-08-13

**Authors:** John Hawthorne, Artūrs Logins

**Affiliations:** 1 University of Zurich, Zurich, Switzerland; 2 Australian Catholic University, Melbourne, Australia; 3 University of Southern California, Los Angeles, USA

**Keywords:** Justification, Gradability, Degrees of justification, Degrees, Credence, Scale, Scalar, Justified, Gradable adjectives, Epistemic justification

## Abstract

The adjective ‘is justified’ has all the hallmarks of a gradable adjective. But the relationship between gradable uses and straightforward predications of the form ‘x is justified’ has been underexplored by epistemologists. In this paper we undertake to do some ground clearing as a prelude to better understanding this relationship.

## Introduction

The adjective ‘is justified’ has all the hallmarks of a gradable adjective. But the relationship between gradable uses and straightforward predications of the form ‘x is justified’ has been underexplored by epistemologists. In this paper we undertake to do some ground clearing as a prelude to better understanding this relationship.

## Evidence for gradability

Linguists use a variety of superficial tests for whether an adjective is gradable. ‘Justified’ passes these tests with flying colours.

*Test 1*. Admissibility of comparative constructions (e.g. ‘x is flatter than y’, ‘x is more regrettable than y’, ‘x is as regrettable as y’ etc.).

Comparative uses of ‘justified’ are not hard to find. (Note that as most of the uses of ‘justified’ in English concern actions we shall freely make use of data from such uses):

1. But the film’s real triumph lies in the fact that the rage effects wear off, meaning bashing your friend’s brains in isn’t quite as justified as it usually is in zombie movies—at least not at first.^1^
2. Over goes Rakitic and the red card that follows is as justified as the challenge was futile and brainless.^2^
3. Prosecutors and judges, for years, have meted out punishment based on a view about how people work and how the world works that is as justified as an argument that a doctor shouldn’t wash his hands before handling a patient.^3^

*Test 2*. Felicity of ‘how F’ questions (e.g. ‘How tall is she?’, ‘How flat is it?’).

Questions of the form ‘How justified is/was x’ are similarly not hard to find:

(4) But how justified were fan fears that Leeds would be starting the new season with a new man in the dugout for the eighth year in a row?^4^
(5) How Justified Is Modi’s New Title?^5^

*Test 3*: Admissibility of degree modifiers (e.g. ‘x isn’t flat at all’, ‘x is extremely regrettable’, ‘x is slightly wet’, ‘x is somewhat bent’, ‘y is completely confident’).

Many degree modifiers are completely felicitous for ‘justified’:

(6) Defence analyst David Perry of the Canadian Global Affairs Institute said those concerns are completely justified given the Trump administration’s penchant for using whatever means necessary to get foreign countries to buy U.S. products.^6^
(7) He also said the government would "ride shotgun" to ensure the €480 m contingency fund is not drawn on unless absolutely justified.^7^
(8) What’s more, that’s perfectly justified by Spain’s clout in Europe at the moment.^8^

Of course, not all degree modifiers are felicitously combined with ‘justified’. For example, ‘x is slightly justified’ and ‘x is very justified’ and ‘x is almost justified’ are pretty marginal to our ear. But it is common even for paradigmatic gradable adjectives to be somewhat choosy about which degree modifiers they admit.^9^ Of course one would ultimately like to know whether there are deep reasons for the infelicity of certain combinations. We will not get to the bottom of that issue here.

## Tallness and justification

For many gradable adjectives, it is very natural to model them in the following way: They are associated with a certain scale and in context, a threshold on the scale is imposed.^10^ This model is, for example, extremely natural for ‘tall’: In context, ‘tall’ is associated with a threshold on the scale of heights. Adjectives for which such a model is appropriate are what Peter Unger and Chris Kennedy (following Unger) call ‘relative gradable adjectives’. It is clear enough that epistemologists who have discussed the relationship between justification and degrees of justification have been conceiving of justification on the model of a relative gradable adjective. Sometimes the imagined threshold is that of coherence, sometimes that of evidential support.^11^ But we do not wish to dwell on such choice points right now. For now, we merely wish to draw attention to a key commonality—that the models in play have a structure akin to those that are typically deployed for relative gradable adjectives. Something like the threshold model is in play in (or is the topic of) the following remarks:

(9) A belief system is justified if it is coherent to a sufficiently high degree. This, in essence, is Laurence BonJour’s 1985 solution to the regress problem. (Olsson 2017)
(10) For any reasonable standard of justification or knowledge, there will be a point at which I just meet, and do not exceed, that standard, and (again assuming I am justified in believing her to be the better arithmetician) I will then not know or be justified in believing the proposition that if she says the sum is wrong, then she is wrong. (Audi 2010: 196).
(11) Compare Peter Klein’s argument (1995, 219). This argument assumes, of course, that degree of justification should be understood as likelihood of truth, and that there is a threshold level of justification required for a belief to be considered ‘justified’. (Huemer 2001: 396, n18)

But there are important differences between ‘tall’ and ‘justified’ that should not be ignored. In particular, ‘justified’ has hallmarks of what Unger and Kennedy call ‘absolute gradable adjectives’, ones which are more naturally associated with endpoints on a scale than thresholds on a scale. In this connection, it bears emphasis that one of the standard tests for absolute gradable adjectives is susceptibility to modifiers like ‘completely’. It is unacceptable to combine such modifiers with ‘tall’—‘John is completely tall’ is utterly infelicitous. However, the expression ‘completely justified’ is altogether natural. Here is a second test from Kennedy: The negation of relative gradable adjectives does not imply their antonyms, while the negative of absolute gradable adjectives does. (‘Not tall’ does not imply ‘short’ while ‘Not pure’ does imply ‘impure’.) It is natural to think of ‘unjustified’ as the antonym of ‘justified’, in which case, this test places ‘justified’ on the absolute side.

Some further data, while admittedly less straightforward tends towards the categorization of ‘justified’ in the absolute category: ‘X is F, but Y is more F/Fer’ sounds considerably more marginal for absolutes than for relatives. Thus while ‘X is tall but Y is taller’ is a very natural everyday use of tall, ‘x is flat but y is flatter’ is a little more awkward sounding. (We don’t want to go so far as to say it is uninterpretable or totally bizarre, merely that it sounds like a slightly more marginal or creative use of language.) And we can report that ordinary informants tended to classify ‘x is justified and y is more justified’ as towards the marginal end. (The tendencies of philosophers were more haphazard. Certain epistemologists we asked were sufficiently imbued with a theory of how justification works as to find ‘x is justified and y is more justified’ to be completely straightforward.) For absolutes, the construction ‘x is F but is not completely F’ tends to be marginal. In line with this, ‘Her suspicion/view/belief that the stock market will crash is justified but not completely justified’ seems pretty marginal.

Kennedy (1999) notes that within the category of absolutes there are two subcategories: Maximal absolutes are associated with the maximal degree of a scale. Meanwhile minimals are associated with the property of having any positive degree whatsoever along a scale. ‘Flat’ is a maximal—it tends to be associated with being maximally flat, while open is minimal—any degree of openness counts as being open. ‘Justified’ patterns more like a maximal. For example, minimals but not maximals combine felicitously with ‘slightly’. But ‘slightly justified’ is extremely awkward. (Alvin Goldman includes ‘slightly justified’ on his list of graded uses of ‘justified’, but that does not at all comport with the habits of English speakers^12^). Similarly, for maximal gradables, ‘x is F but could have been more F’ is pretty marginal (Kennedy notes for example that ‘X is straight but could have been made straighter’ is a bit odd). In line with this, an ‘The stockbroker was justified in thinking that the stock market would crash but could have been more justified’ is also pretty marginal.^13^

We submit that the absolute features of ‘justified’ should at least give one pause when it comes to the threshold model.

## Justification and probability

As an instructive case study, it is useful to see how problematic it is to try to model the behavior of ‘justified’ using a probability scale. The idea that degrees of justification are somehow a matter of epistemic probability is certainly a common idea in the literature:

(12) To express the common view a little more precisely, the degree of justification for accepting the proposition *h* given the evidence *e* (based on the background assumption *b*—this is suppressed in the following discussion) is the conditional probability of *h* given *e*, or *P*(*h*|*e*). (Shogenji 2012: 30).

See also Huemer on Klein above (cf. (11)).

The basic idea of such models is that one will have more justification to believe P than Q just in case P is more probable than Q. This model can be developed in a way that models ‘justified’ on ‘tall’ so that ‘x has justification to believe Q’/‘is justified in believing Q’ just in case P surpasses a probabilistic threshold set by context. But one might also be motivated by the considerations in the previous section to instead develop the model in such a way that ‘justified’ gets associated with an endpoint on the probabilistic scale, so that, strictly speaking ‘x is justified in believing p’ requires maximal probability.

But whichever way one goes, there are reasons to doubt that such a model is appropriate. One thing to realize of course is that this model can at best be a model of how *propositional* justification for belief works. The literature distinguishes *doxastic* justification from *propositional* justification—one can have great evidence for P but one’s belief be in poor standing—and hence doxastically unjustified—because one’s belief is not based on one’s good evidence but on a bad argument instead. And even more obviously this model is not well suited to explaining degrees of justification for fear, concern, suspicion, suspending judgment and so on. Additional justification for suspending judgment is not tantamount to additional epistemic probability. (Distinguishing ‘epistemic’ from ‘non-epistemic’ uses of ‘justified’ will not sidestep the issue: degree of justification for suspending judgment seems to be an epistemic issue.)

But even focusing just on the case of justification to believe, there is good reason for doubting that degrees of justification correspond to degrees of likelihood.

First, note that it is perfectly felicitous to say that an action or belief or emotion is not justified at all. And a natural hypothesis is that ‘not justified at all’ signifies zero degree of justification. If degrees of justification corresponded to epistemic probabilities, the natural hypothesis would be that belief in a certain proposition P is not justified at all just in case its epistemic probability is zero. But that hypothesis is not borne out. One is not justified at all in believing that coin will come up heads—but the epistemic probability of that outcome is intermediate, not zero. This consideration is not decisive however: for example, perhaps being justified at all requires surpassing a threshold on the probability scale (we shall return to ‘at all’ presently).

But note second, and more decisively, that ‘justified’ does not felicitously combine with numerical (e.g. ratio and proportional modifiers) modifiers like ‘twice as’, ‘ten percent’ and so on.^14^ If justification was linked to a scale like probability that is friendly to numerical modifiers, then one would expect much more felicity than we do in fact see.

A comparison with ‘being confident’ is somewhat illuminating here. ‘Confident’ has notable similarities and differences with ‘justified’. Like justified it seems to pattern like an absolute gradable: ‘Completely confident’ is felicitous, ‘confident but not completely confident’ is marginal, and so on.

Moreover, there are analogous points to be made as regards ‘not being justified at all’ and ‘not being confident at all’. Just as we can say a belief that a die will come up 3 is not justified at all, it is similarly natural to say that one is not confident at all that the die will come up three. But in this case, one’s credence is far from zero. (This point is noted by Williamson, *mn*.) Thus, the flatfooted idea that one is not confident at all is tantamount to zero credence is wrongheaded.

But in another respect the behavior of ‘confident’ is quite different to ‘justified’. And this is because, even outside the philosophy room, numerical constructions combine felicitously with ‘confident’. Thus, it is not at all unnatural to say that ‘I am three times more confident that the coin will come up heads than that the die will come up three’ and ‘I am fifty per cent confident that the coin will come up heads’.^15^

This creates a prima facie puzzle. How can it be that one can truly say ‘I have no confidence/I am not confident at all that she will win the bet’ while also finding it natural to say ‘I am three times more confident that she will win the bet than that the die will come up three’?

A few solutions suggest themselves. One view is that two quite different scales are in play in the context of each of the speeches which have little to do with each other. According to an alternative and arguably more plausible view, there is a single scale, where ‘being confident at all’ marks not an endpoint on the scale but a threshold. A natural first pass take on ‘being confident at all’ is along the lines of ‘being at all close to complete confidence’. (In a sense this introduces a second scale—degrees of closeness to the max—but it is highly parasitic on the original probability scale.)

It is worth noting in passing that similar ‘at all’ data arises for other gradable absolutes. Thus ‘not being flat at all’ and ‘not being dry at all’ do not plausibly equate to being maximally bumpy and wet. In those cases, a threshold approach naturally suggests itself and it is natural to extend the threshold approach to ‘confident’.

In short, the numericals make it much more natural to associate ‘confident’ with something that has the structure of a probability scale that it is for ‘justified’. For confident, there is a reasonable prima facie case that ‘at all’ marks a threshold on the scale corresponding to something like ‘at all close to the max’.

Many philosophers will think something has obviously gone wrong. On the picture presented ‘confident’ and ‘completely confident’ is associated with the maximal end of the credal scale so that ‘completely confident’ is equivalent to subjective certainty. They will complain that it is obvious that one can be completely confident in the ordinary sense without being certain (and even more obvious that ‘confident’ in the ordinary sense does not equate to certainty). But this is too quick. First, it is worth noting that in many languages ‘confident’ and ‘certain’ get translated the same. Thus, in Latvian both ‘X is confident that p’ and ‘X is certain that p’ are naturally translated as ‘X ir pārliecināts, ka p’ (similarly, in Russian both ‘confident’ and ‘certain’ can be translated by ‘yвepeн’). Second, note that analogous issues arise for other gradable adjectives. A philosopher contrives a demanding sense of being empty of beer and then notes that in the ordinary sense ‘being completely empty’ can be truly ascribed to a beer glass in a pub even though the glass is not empty in the demanding sense. There are well known ways to approach this. One might say that the pub ascription is not strictly speaking true but nevertheless felicitously assertable and explain why. Or one might introduce a kind of contextualism according to which various scales are in play in contexts and that certain tiny quantities of beer don’t register on the scale in play at certain contexts (cf. Greco 2015).

But third we should note an extra complication. As Chris Kennedy notes, once we have a scale with a maximum and a minimum, adjectives often exhibit a degree of flexibility as to whether they gravitate toward a reading that means something like ‘maximum’ or instead to something like ‘not minimum’. He illustrates this for ‘opaque’ and ‘transparent’:

(13) The antonyms *opaque* and *transparent* verify this prediction. According to the diagnostics discussed above, these use a totally closed scale (*completely*/*slightly opaque*/*transparent*), and so are in principle compatible with either minimum or maximum standard interpretations in the positive form. The following examples show that both interpretations are in principle possible. Consider a context in which I am manipulating a device that changes the degree of tint of a car window from 0% (completely transparent) to 100% (completely opaque). (67a) can be felicitously utterered at the point at which I have almost reached 100% of tint, demonstrating both that *opaque* can have a maximum standard (I am denying that the glass is completely opaque) and that *transparent* can have a minimum standard (partial transparency).
  (67) a. The glass is almost opaque, but not quite. It’s still transparent.
  b. The glass is almost transparent, but not quite. It’s still opaque.
  Likewise, (67b) can be used to describe the reverse situation: one in which I have dialed down almost to 0% of tint. Here *transparent* has a maximum standard (complete transparency) and *opaque* has a minimum standard (partial opacity). (Kennedy 2007: 37)

And:

(14) [F]or any closed scale adjective, a maximum standard interpretation entails a minimum one, but not vice-versa. Assuming that stronger meanings are in general favored […], this preference follows. If minimum standard interpretations were impossible, however, then the second sentences in (67a,b) would be contradictory. (Kennedy 2007: 37–38).

What we wish to point out is that once we have an ‘at all’ threshold—where everything under the threshold is not at all close to the maximum, then we have in effect a closed scaled with the maximum at one end and the ‘at all’ boundary at the lower end. And if Kennedy is right, there is the prospect that ‘confident’ may oscillate somewhat between the maximal and minimal interpretations.^16^ This point generalizes. Insofar as ‘justified at all’ and ‘completely justified’ are associated with a closed scale, there is the prospect that ‘justified’ may oscillate somewhat between maximal and minimal interpretation. We leave further exploration of this issue to another occasion.

## Scale-primacy and scale-derivativeness

For some expressions, it is plausible that some grasp of a scale is integral to understanding them. For example, it is plausible that an understanding of ‘heavy’ and ‘tall’ requires some grasp of the scales of weight and height respectively. But it is important to realize that even when scales are completely irrelevant to the understanding of a predicate, comparative uses of the predicate can be introduced in a derivative way. Consider, for example, ‘is a feast’. It is easy enough to get the hang of such constructions as ‘That meal was more of a feast than that one’. One particularly natural way to understand such claims is as tantamount to ‘That meal was closer to being a feast to that one’ where there is a rough and ready grasp of some scale of closeness. Of course, grasp of a closeness scale of this sort is hardly integral to one’s grasp of ‘is a feast’. Nevertheless, having mastered that predicate one might, often on the fly, contrive some sense of comparative closeness to being a feast. Consider similarly ‘circular’. It is not plausible that mastering a circularity scale is integral to understanding ‘being circular’. But having mastered ‘circular’ it is not hard to contrive a rough and ready scale of being close to being circular. The scales thus constructed will typically reflect some rough and ready sense of how close things are to the phenomenon in question. Our ability to do this is quite general. The claims ‘That is more of an omelet than that’ and ‘She is more blameless than him’ are perfectly interpretable—each reflects the speaker’s rough and ready sense of comparative closeness to being an omelet and being blameless respectively.

Our general ability to introduce comparative structures along these lines should allay any suspicion that talk of degrees of justification is nonsense. Consider Jonathan Sutton (who thinks that a belief is justified if and only if it is known):

(15) For me, the notion of a belief being *more* justified than another justified belief is, strictly speaking, nonsense—knowledge does not come in degrees. (Sutton 2007: 154, n4.)

This seems excessive.^17^ We have a general ability to contrive rough and ready scales of closeness to any given phenomenon and to use graded adjectives to express that scale.^18^

The charge of nonsense is thus unlikely to prove well founded. But some interesting questions remain. A better question than whether graded uses of ‘justified’ express sense or nonsense is the question whether such uses are derivative in the way ‘being more of a feast’ is. On one picture, the non-graded notion of ‘justified’ is primary and the graded use highly derivative. On this picture the basic semantics of ‘justified’ does not associate it with a scale but we can nevertheless “coerce” a scalar use out of it by using general linguistic mechanisms that allow us to generate a scalar use out of expressions that do not get associated with a scale via our foundational understanding of them. On a second picture, some kind of scale is integral to understanding ‘justified’. Call the latter view a ‘scale-fundamental’ view of ‘justified’ and the former a ‘scale-derivative’ view. Which view is correct? A second question is also worth addressing: Is there anything of potential significance to philosophers that may be at stake here? We address each question in turn.

We don’t propose to offer a definitive verdict on whether ‘justified’ is scale-derivative or not. But we would like to present two interesting lines of thought that militate in the direction of scale-derivativeness.

One line of thought worth drawing attention to is presented in Burnett’s (2017) comprehensive treatment of gradable expressions. There she focusses on what she calls a ‘paradox of absolute adjectives’ (Burnett 2017: 70). The paradox can be usefully represented as an apparently inconsistent triad: (1) Absolute adjectives are gradable. (2) Gradability is derived from context-dependent sensitivity to comparison classes. But (3) The semantic denotation of absolute adjectives has nothing to do with comparison classes.

Burnett in effect says the triad is not inconsistent. The semantic denotation of absolute adjectives does not have gradability associated with their denotations. Nevertheless, we can “stretch” the meaning of the relevant adjectives in order to coerce a gradable use out of them:

(16) I will show that, through giving an appropriate tolerant and strict semantics for AAs [i.e. absolute adjectives], we can arrive at an understanding of how it is possible to “stretch” the meaning of an absolute term to... take in the gradable gamut of reality. (Burnett 2017: 72).

In short, (3) is correct, but that does not deprive absolute adjectives of a gradable use, since it is the coerced/stretched meaning that is responsible for such a use. (2) can be preserved alongside (1) by insisting that the stretched meaning involves sensitivity to comparison classes and that it is this stretched meaning that accounts for (1).

This is not the place to rehearse Burnett’s admirably detailed case for her picture of absolute adjectives. What bears emphasis in this context is that her treatment entails that *all* absolute adjectives are scale-derivative. Within her framework, the fact that ‘justified’ is absolute is a decisive reason for classifying it as scale-derivative.^19^

Here is a second prima facie reason for thinking that ‘justified’ is scale-derivative. It is tempting to think that ‘justified’ has a strong affinity with such normative concepts as that of permissibility and blamelessness. This is a theme of work in progress by one of us (NN). It is also a theme of some interesting literature on justification. For example, Beddor (2017), distinguishes strong necessity modals like ‘must’ from weak necessity modals like ‘ought’, arguing that ‘justification’ functions as the dual of a weak necessity modal, where the semantic value of that dual is appropriately glossed as *faultlessness*.^20^ Here is not the place to defend views of this kind. But it is worth pointing out that if they are right, that may help the case in favor of the scale-derivative view. After all graded uses of ‘faultlessness’—“That was more faultless than that’—or of ‘permissibility’—‘That was more permissible than that’—seem to be fairly paradigmatic cases of coerced, scale-derivative, uses of adjectives.^21^

So much for our first question. What of our second? Is there anything of philosophical interest at stake when it comes to scale-fundamentality or scale derivativeness? Here we wish to make two observations.

First, in cases of adjectives for which graded uses are scale-derivative, we should be sceptical of any kind of conceptual analysis of that adjective (or the concept associated with it) that appeals to a scale, at least insofar as the project of conceptual analysis is supposed to make vivid what our basic understanding of the adjective consists in. After all, if the adjective is scale-derivative, then associations with a scale would appear to be a secondary, coerced phenomenon, and peripheral to the basic understanding of a term. This raises an additional prima facie concern (to the one raised earlier) about such analyses in the literature as Olson’s gloss on Bonjour (cited earlier), at least insofar as such claims are offered in anything like the spirit of a conceptual analysis.^22^

(9) A belief system is justified if it is coherent to a sufficiently high degree.[…] (Olsson 2017).

Our second observation is that in many cases, scale-derivativeness brings with it a significant variability in the scale associated with graded comparative adjectives, and there may be good reason to expect such variability in the case of the graded use of ‘justified action’ and ‘justified belief’. Let us elaborate. When it comes to coerced, scale-derivative uses, there will be no guidance concerning which scale to deploy that emanates directly from the initial semantical denotation, since that semantical denotation does not encode a scale. In some cases, however, there will be a salient, natural way to construct a scale on the fly. When this is the case, we might expect quite a bit of constancy in the way a scale is generated from context to context. But when this is not the case, we should expect quite a bit of unruliness in the behavior of graded uses from conversation to conversation. Let us begin with an illustration of the former, more disciplined, pattern. Suppose we think of permissible credences in a proposition P as forming a subrange of the standard credence scale. Perhaps, for some P, it is the range 0.2–0.3. Then there is an overwhelmingly natural way of associating a scale with ‘credence x is more permissible than credence y’: we treat the credences in the permissible range as completely permissible and then rank individual credences outside the range as more or less permissible according to how close they are to the range. Because of the naturalness of this way of doing things it would take a fair bit of effort to imagine a context where we say ‘The credences that are completely permissible are 0.2–0.3 but the credence 0.4 is more permissible than 0.32’.

The credence case is relatively special, however. For many coerced uses there is no particularly salient natural scale to turn to when contriving a scale, and whichever scale is constructed on the fly will be highly sensitive to the goals and background of the conversation in which the coerced use is in play. Consider for example ‘x is more of a feast than y’. There are various parameters that might be brought into play: The variety of food involved, the quantity of food involved, the fanciness of the food involved, or some vague weighting of some or all of these factors. By contrast with ‘permissible credence’ we would expect significant variability in the coerced scales.

What of ‘justified’ as applied to either actions or beliefs? Here again, analogies with permission and or faultlessness may be instructive. Is there a salient natural way of ranking actions that are not faultless as more or less faultless (or of ranking actions that are not permissible as more or less permissible)? As far as we can tell, the situation seems rather more like that of a feast than that of permissible credence. One might contrive a rough and ready way of counting faults, and then rank actions as more or less faultless dependent on the number of faults. Or one might contrive a rough and ready way of weighing seriousness of faults and count actions as more or less faultless dependent on the “seriousness” scale. Or one might contrive a rough and ready way of ranking how aware people were of the fact that were at fault and use that. But there doesn’t seem to be a very salient and natural scale to gravitate to.^23^ And as far as we can see, while there may be a salient scale for permissible credence, the situation for permissible and/or faultless belief seems to be rather like that of permissible and or faultless action. Among beliefs that are at fault, epistemically speaking, how do we rank them for being more or less faultless? It would, after all, be a bit naïve to think that we can grade on a scale of “probability on the evidence”. After all, it is a familiar point that beliefs well supported by the evidence may nevertheless be formed by imperfect means. Furthermore, we have seen that graded uses of ‘justified belief’ do not play with numericals in the way one might expect if it were associated with a probability scale. Moreover, those of us who are happy to think that a belief can be knowledge without being based on evidence might think that an evidentially unsupported belief that is not knowledge might nevertheless in some sense count as being close to being justified simpliciter on account of there being a nearby situation where the same method produces knowledge. Our suspicion, then, is that there may not be any natural scale to turn to when generating a coerced meaning for ‘That belief is more justified than that’. And if that is right, we should place graded uses of ‘justified’ in the ‘highly variable’ category.

If graded uses of ‘justified’ are both scale-derivative and rather unruly, this can serve as a partial vindication of what has gone on in epistemology. Epistemologists have for the most part tended to say a lot about what it takes for a belief to be justified and have said very little about graded justification. If the graded uses are scale-derivative then it is best to ignore graded uses when providing an account of justification simpliciter.^24^ And if the graded uses are unruly, displaying significant variation in content from conversation to conversation, then there may be little gained by tackling a general question of the form ‘What is it for a belief to be more justified than another?’ Perhaps epistemologists can proceed as they were without undue concern.

## Footnotes

1 See: https://bloody-disgusting.com/editorials/3562284/needs-enemies-friends-like-10-bonds-bred-malice-movies/.

2 See: https://www.telegraph.co.uk/football/2016/04/30/real-betis-v-barcelona-la-liga-title-race-live/.

3 See: https://abovethelaw.com/2015/07/why-judges-and-prosecutors-dont-care-if-theyre-right/.

4 See: https://www.leeds-live.co.uk/sport/leeds-united/inside-story-marcelo-bielsas-contract-16345707.

5 See: https://www.youthkiawaaz.com/2019/05/the-divider-in-chief-of-india-times-version/.

6 See: https://www.theglobeandmail.com/canada/article-new-canadian-fighter-jets-will-need-us-certification-dnd-2/.

7 See: https://www.rte.ie/news/politics/2019/0516/1049941-bruton-defends-rejection-of-warning-over-broadband-plan/.

8 See: https://www.theguardian.com/world/2019/may/30/spain-pedro-sanchez-victorious-acting-pm-europe-elections.

9 For instance: while ‘very tall’, ‘completely pure’, ‘slightly wet’ are all felicitous, ‘very pure’, ‘completely tall’, and ‘slightly small’ appear to be infelicitous.

10 Note that we do not say ‘semantically associated’. As we shall explain in the final section, it is not true in general that scalar uses are explained by semantic association with a scale.

11 In this paper we are largely operating with the kind of framework introduced by Chris Kennedy and which has been most dominant in work by philosophers of language on gradable adjectives (see, for example, Kennedy 1999, 2007; Kennedy and McNally 2005). However, the degree-theoretic framework for handling gradable adjectives has not been beyond criticism in the semantics literature. See, notably, recent work by Wellwood—e.g. Cariani et al. (*ms*) and Wellwood (2015). It is a good question to what extent the ideas in this paper can happily be adapted to Wellwood’s alternative framework. It is beyond the scope of this paper to pursue that issue. For other critical material on Kennedy see Lassiter and Goodman (2014, 2017).

12 See, Goldman: “Support for reliabilism is bolstered by reflecting on degrees of justifiedness. Talk of justifiedness commonly distinguishes different grades of justifiedness: ‘fully’ justified, ‘somewhat’ justified, ‘slightly’ justified, and the like.[..] These distinctions appear to be neatly correlated with degrees of reliability of belief-forming processes.” (Goldman 1986: 104).

13 Kennedy also seems to think that when a gradable adjective ‘is F’ is associated with a scale that has a closed upper bound, the gradable adjective will tend to be a maximal absolute. As Daniel Lassiter has noted this is dubious. The expression ‘is likely’ is not absolute but plausibly it is associated with a probability scale with a closed upper bound, see Lassiter (2017: 82ff).

14 The data from usage is quite dramatic here. A google search revealed 6 and 1390 hits respectively for ‘fifty percent justified’, as contrasted with ‘fifty per cent confident’, and 0 and 1320 hits respectively for ‘ninety percent justified’ and ‘ninety per cent confident’. Meanwhile, ‘twice as justified’ and ‘twice as confident’ got 6 and 61,300 hits respectively.

15 As a referee noted, this contrast between ‘confident’ and ‘justified’ may have its roots in the fact that ‘justified’ has a deontic use whereas ‘confident’ does not. When we say that a coach was justified in using a formation, we seem to be somehow conveying the faultlessness or permissibility of the act. The fact that the concepts of faultlessness and permissibility do not play at all well with ratio modifiers may thus be intimately connected to relevant behavior of ‘justified’. For more on the normative import of ‘justified’, see Beddor (2017).

16 For more on the dual life of ‘confident’ see Logins 2019. For an exploration of another epistemically significant gradable adjective that seems to exhibit a maximal only interpretation, namely ‘supported’, see Logins 2020.

17 Unless, that is, we understand ‘strictly speaking nonsense’ to merely entail that the original semantic denotation of ‘justified’ didn’t already bring a scale with it. Notice though that this is completely compatible with the claim that sentences of the form ‘That belief is more justified than this’ express a proposition, are truth evaluable and so on. Relevant here is our discussion of Burnett below.

18 Of course, scales will in some cases be more salient than others. It is often quite easy to rank things for how circular they are. But the ability to rank meals for closeness to a feast may be rather more messy as there may be less of a reasonable expectation of a shared sensibility as regards closeness ranking.

19 Note that the scale-fundamental adjectives that we earlier offered as paradigmatic—‘tall’ and ‘heavy’—are not absolutes.

20 He suggests further that the permissibility gloss isn’t quite right, since that would wrongly treat the property of being justified as the dual of a strong necessity modal. Note that the argument of the present paragraph would be weakened if, owing to the polysemy of ‘justified’, only *some* uses of ‘justified’ involved a systematic connection with faultlessness. After all, as a referee pointed out, different meanings associated with a single polysemous expression might recruit different tools.

21 We say ‘help the case’ but we should demur from anything stronger than this. After all, as a referee pointed out, it is not inconceivable that two expressions are equivalent but nevertheless recruit different technologies (so that one is scale-derivative and the other not).

22 Interestingly, this also raises questions about an approach to (arguably related) normative concepts like that of permissibility that has been made standard by work of Kratzer (1977, 1991). On that approach permissibility typically understood in terms of quantification over a relevant domain of situations (a modal base) that gets a ranking (via an “ordering source”). Insofar as the ordering source is supposed to represent the underlying semantic workings of ‘ought’ and ‘may’, we may worry that this improperly treats certain graded talk as scale-fundamental when in fact it is scale-derivative. For a critique of the ordering source approach to ‘Ought’, see Finlay (2016). An aspect of Beddor (2017) that we are less comfortable with is its central reliance on a ranking of situations by a norm. It is beyond the scope of this paper to pursue this issue further.

23 Clearly, ranking things as more or less along a scale need not bring with it congruence verdicts that permit judgments along such lines as ‘The distance between x and y is the distance between y and z’. And without congruence verdicts, there will be no sound basis for such claims as ‘That is three times more F than that one’. Neta (2015) doubts whether departures from rational permissibility can be measured. He writes: “To say that some cognitive options depart more or less from rational permissibility than others is not to imply that departures from rationally permissibility can be measured, any more than saying that some people are more scrupulous than others implies that scrupulousness can be measured. Comparisons of more or less can be truthfully made, even when such comparisons cannot be measured. (The comparative relation could, for instance, generate only a partial ordering among relata.)” (Neta 2015: 288). We think that the best way of precisifying this thought is in terms of an absence of congruence structure.

24 Notice also that if graded uses are unruly in the way described, then those philosophers who (by our lights incorrectly) proceed as if justification simpliciter were somehow to be analysed in terms of a scale of justification would be all over the place in regards to which kind of analysis they settle upon. And this predicted divergence is arguably precisely what we find.
